# Consequences of a Maternal High-Fat Diet and Late Gestation Diabetes on the Developing Rat Lung

**DOI:** 10.1371/journal.pone.0160818

**Published:** 2016-08-12

**Authors:** Michelle L. Baack, Benjamin J. Forred, Tricia D. Larsen, Danielle N. Jensen, Angela L. Wachal, Muhammad Ali Khan, Peter F. Vitiello

**Affiliations:** 1 Children’s Health Research Center, Sanford Research, Sioux Falls, SD, United States of America; 2 Department of Internal Medicine, Sanford School of Medicine-University of South Dakota, Sioux Falls, SD, United States of America; 3 Department of Pediatrics, Sanford School of Medicine-University of South Dakota, Sioux Falls, SD, United States of America; 4 Children’s Health Specialty Clinic, Sanford Children’s Hospital, Sioux Falls, SD, United States of America; Johns Hopkins University, UNITED STATES

## Abstract

**Rationale:**

Infants born to diabetic or obese mothers are at risk of respiratory distress and persistent pulmonary hypertension of the newborn (PPHN), conceivably through fuel-mediated pathogenic mechanisms. Prior research and preventative measures focus on controlling maternal hyperglycemia, but growing evidence suggests a role for additional circulating fuels including lipids. Little is known about the individual or additive effects of a maternal high-fat diet on fetal lung development.

**Objective:**

The objective of this study was to determine the effects of a maternal high-fat diet, alone and alongside late-gestation diabetes, on lung alveologenesis and vasculogenesis, as well as to ascertain if consequences persist beyond the perinatal period.

**Methods:**

A rat model was used to study lung development in offspring from control, diabetes-exposed, high-fat diet-exposed and combination-exposed pregnancies via morphometric, histologic (alveolarization and vasculogenesis) and physiologic (echocardiography, pulmonary function) analyses at birth and 3 weeks of age. Outcomes were interrogated for diet, diabetes and interaction effect using ANOVA with significance set at p≤0.05. Findings prompted additional mechanistic inquiry of key molecular pathways.

**Results:**

Offspring exposed to maternal diabetes or high-fat diet, alone and in combination, had smaller lungs and larger hearts at birth. High-fat diet-exposed, but not diabetes-exposed offspring, had a higher perinatal death rate and echocardiographic evidence of PPHN at birth. Alveolar mean linear intercept, septal thickness, and airspace area (D_2_) were not significantly different between the groups; however, markers of lung maturity were. Both diabetes-exposed and diet-exposed offspring expressed more T1α protein, a marker of type I cells. Diet-exposed newborn pups expressed less surfactant protein B and had fewer pulmonary vessels enumerated. Mechanistic inquiry revealed alterations in AKT activation, higher endothelin-1 expression, and an impaired Txnip/VEGF pathway that are important for vessel growth and migration. After 3 weeks, mortality remained highest and static lung compliance and hysteresis were lowest in combination-exposed offspring.

**Conclusion:**

This study emphasizes the effects of a maternal high-fat diet, especially alongside late-gestation diabetes, on pulmonary vasculogenesis, demonstrates adverse consequences beyond the perinatal period and directs attention to mechanistic pathways of interest. Findings provide a foundation for additional investigation of preventative and therapeutic strategies aimed at decreasing pulmonary morbidity in at-risk infants.

## Introduction

Diabetes and obesity during pregnancy are escalating at an astounding rate [1], and consequences extend beyond those of the mother to the developing fetus [2, 3]. Along with other morbidities, infants born to diabetic mothers (IDMs) have a higher rate of respiratory distress [4–6], persistent pulmonary hypertension of the newborn (PPHN) [6–8], and perinatal mortality [9]. Historically, these consequences have been attributed to hyperglycemia-induced delay in fetal lung maturation and surfactant protein production, which are well-recognized complications of a diabetic pregnancy [6, 10–13]. However, even when fetal lung maturity testing is normal, IDMs remain at higher risk for pulmonary complications [14], which suggests additional pathogenesis. Moreover, maternal obesity, even in the absence of hyperglycemia, is an independent risk factor for respiratory disease (6, 8, 17) and PPHN (6, 8, 18), which implicates a role for additional circulating fuels, including lipids (19). The objective of this study was to determine the effects of a maternal high-fat (HF) diet, alone and alongside late-gestation diabetes, on lung alveologenesis and vasculogenesis, and to ascertain if consequences persist beyond the perinatal period.

This study investigates four relatively understudied questions about fuel-mediated effects on fetal lung development. First, can maternal diabetes that begins *after* primary organogenesis alter structural lung development? Previous animal studies demonstrate that exposure to hyperglycemia at conception or early in pregnancy impairs alveolar development [13, 15, 16]. However, whether structural pulmonary changes can occur from exposure to maternal diabetes late in gestation is relatively unknown. Second, can a maternal HF diet, alone or alongside late-gestation diabetes, increase pulmonary morbidity in the developing offspring? It is increasingly evident that pulmonary morbidities found in infants born to both diabetic and obese mothers overlap significantly [6, 8, 17], so there is growing interest in additional fuel-mediated offenders, including lipids [18, 19]. Third, can exposure to excess circulating maternal fuels influence pulmonary vascular development? It is known that alveolar development is reliant on normal vascular signaling [20, 21]. Additionally, IDMs demonstrate impaired transitional pulmonary hemodynamics [22], and autopsies of premature infants born to diabetic mothers have demonstrated increased muscularization of small pulmonary arteries [7]. This suggests that abnormal vascular development could play a role in pulmonary disease. However, the effects of excess circulating fuels on pulmonary vasculogenesis are relatively understudied. Fourth, can prenatal exposure to maternal diabetes or HF diet lead to pulmonary morbidity beyond the perinatal period? There is growing evidence to suggest a link between metabolic derangements in lipid and glucose metabolism and asthma [23], and infants born to women with an elevated body mass index during pregnancy are at increased risk of wheezing [17] later in life.

To date, there are very few studies investigating the effects of maternal HF diet on pulmonary development [6, 24], and to our knowledge, no other study has looked at the combined effect or followed offspring past the perinatal time-point. Given the assumption that both maternal diabetes and a HF diet independently impair lung development [15, 24], it is likely that overlapping and allosteric perturbations in the maternal-placental-fetal environment (such as fetal hyperinsulinemia, inflammation, etc) would contribute to worsening pathogenesis. Gestational diabetes and obesity are often co-morbid conditions in child-bearing women [25]. Indeed, 76% of women with gestational diabetes are overweight or obese [26]. Therefore, delineating the combined effect is important. This study uses a rat model to determine the effects of a maternal HF diet, alone and alongside late-gestation diabetes, on offspring’s pulmonary alveologenesis and vasculogenesis using morphometric, histologic and physiologic (echocardiography, pulmonary function) analyses at birth and three week time points and uncovers additional mechanistic leads through further inquiry of key molecular pathways.

## Materials and Methods

### Animal care

**Fig 1:**
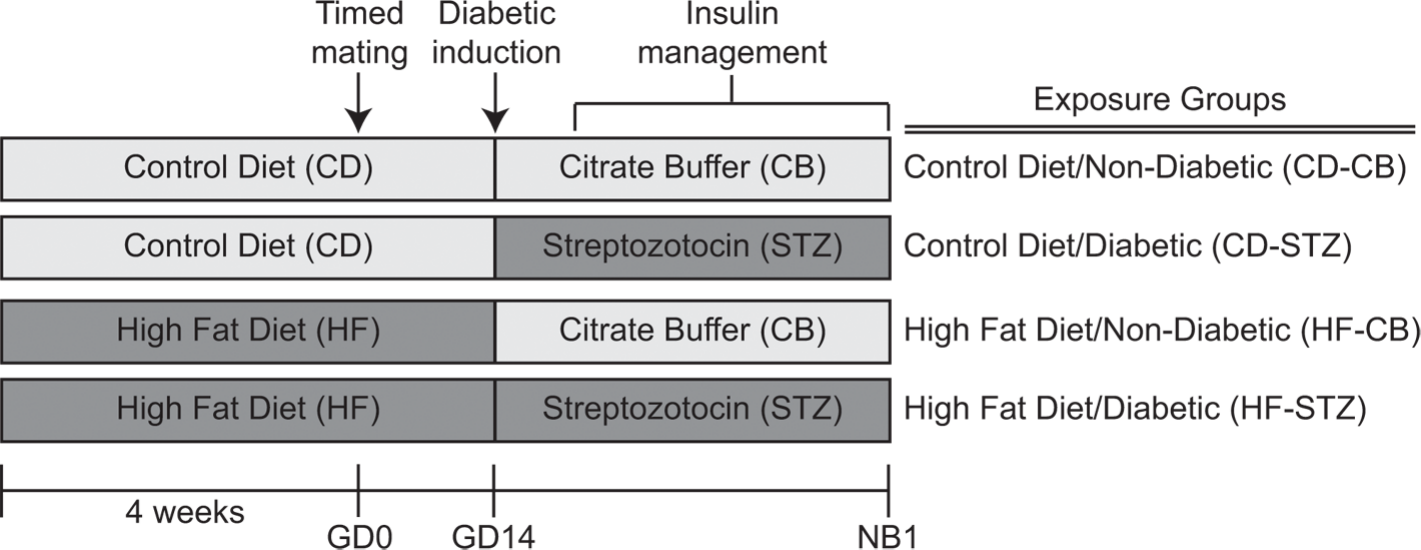
Experimental paradigm. Female Sprague Dawley rats were fed either control (CD) or high-fat (HF) diet at least 4 weeks prior to mating and bred with normal males. A positive vaginal swab for spermatozoa was deemed gestational day zero of pregnancy (GD0). On GD14, dams were injected with either citrate buffer (CB) vehicle or streptozotocin (STZ) to induce diabetes, which was controlled with sliding scale insulin twice daily. Offspring from four groups were analyzed on day one of life (NB1) or cross fostered to normal dams and analyzed at 3 weeks of age.

The study followed guidelines set forth by the Animal Welfare Act and the National Institutes of Health Guide for the Care and Use of Laboratory Animals and was under approval from the Sanford Research Institutional Animal Care and Use Committee. As previously described in detail [27] and outlined in Fig 1, female Sprague-Dawley rats (Harlan Laboratories Inc., Indianapolis, IN) were assigned either control (CD—18% calories as fat) or high-fat diet (HF—40% of calories as fat) prior to timed breeding. On gestational day (GD) 14, the pregnancy was confirmed by ultrasound, and dams received either 0.09 M citrate buffer (CB) or 65 mg/kg of intraperitoneal streptozotocin (STZ) (Sigma Life Sciences, St. Louis, MO) in CB to induce diabetes. Thereafter, two daily glucose concentrations and daily β-hydroxybuterate levels were measured by tail nick sampling, and sliding scale, subcutaneous insulin was administered to keep glucose levels in a target range of 200–400 mg/dl and ketosis to a minimum. Dams typically delivered on GD22, yielding newborn (NB1) offspring from 4 distinct groups: controls (CD-CB), diabetes-exposed (CD-STZ), diet-exposed (HF-CB) and combination-exposed (HF-STZ) as outlined in Fig 1.

To decrease confounding from postnatal influences, such as feeding and rearing from diabetic or HF-fed mothers, litters were culled to equal size (6 pups), and pups were cross-fostered to normal mothers for evaluation at later time-points. In order to determine true litter size and perinatal mortality, delivering dams were sacrificed after cross-fostering for post-mortem evaluation of both uterine horns. Live vs. dead offspring could then be accounted for by counting obvious placentation points alongside the number of live pups, retained still births and dead pups found in the cage. All animals, including dying pups, met humane endpoints in as timely a manner as possible. Euthanasia occurred under isofluorane:oxygen induction followed by cervical dislocation for newborn pups and cardiac puncture/heart removal for older animals.

### Serum analysis

Whole blood was collected from non-fasting dams at baseline, after 28 days of specified diet, at GD14 and at post-partum time points for analysis as previously described [27]. In short, serum triglycerides (TG) were measured using a Triglyceride Colormetric Assay Kit (Thermo Fisher Scientific Inc. Waltham, MA). Serum insulin, c-peptide and leptin levels were detected with the Milliplex (Billerica, MA) MAP Rat Metabolic Panel, and IL-6 and TNFα were detected with the Milliplex MAP Rat Cardiovascular Disease panel according to manufacturer’s directions and analyzed using the Luminex 200 Milliplex Analyzer (EMD Millipore; Billerica, MA).

### Lung Histology

For all histologic analyses, lungs were inflation fixed through uniform tracheal instillation of 10% neutral buffered formalin and were processed on a Leica 300 ASP tissue processor. The paraffin embedded tissues were sectioned at 5μm, and slides were prepared for further processing as indicated.

Measurements for mean linear intercept, septal thickness, and airspace area (D_2_) were performed on hematoxylin and eosin-stained lung sections using a grid overlay consisting of parallel lines spaced 50μm apart. Mean linear intercepts and septal thickness were directly measured using an automated ImageJ macro (NIH, Bethesda, MD) in accordance with quantitative assessment recommendations made by the American Thoracic Society [28, 29]. Similarly, measurements of septal thickness were conducted with the exception that appropriate thresholding marked the interstitial tissue and not the alveolar space. Measurements for alveolar area (D_2_) were completed as previously described [30].

Proliferating cells were stained using anti-Ki67 (1:1000, Biocare Medical). For TUNEL staining, the TACS-XL In Situ DAB Apoptotis Detection Kit (Trevigen Inc., Gaithersburg, MD) was used according to the manufacturer’s protocol. Trichrome staining with hematoxylin, biebrich scarlet/acid fuchshin solution, phosphomolybdic/phosphotungstic acid and aniline blue was performed to detect significant fibrosis. To qualitatively evaluate glycogen deposition as a marker of lung maturity, Periodic acid-Schiff (PAS) staining was performed with 1% periodic acid, followed by Schiff solution and hematoxylin counterstain. Lung vessels were stained using anti-vWF (1:250, Abcam, Cambridge, MA) with hematoxylin counterstaining. For antibody optimization and final staining, the Benchmark XT automated slide staining system was used (Ventana Medical Systems, Inc.) with the Ventana iView DAB detection kit. Exclusion of the primary antibody served for negative controls. Images were visualized and captured at 2x - 60x with a Nikon P90i microscope and analyzed using NIS-Elements software (Nikon Instruments Inc., Melville, NY). The number of vWF-positive vessels per field was compared using the average of five fields per lung section.

### Physiology

Pulmonary function testing was performed using a forced oscillation technique with module FX4 of the FlexiVent FX system (Scireq, Montréal, Canada). Three week-old rats were anaesthetized with 100 mg/kg ketamine and 10 mg/kg xylazine followed by a tracheotomy inserting an 18 gauge blunt-end catheter for mechanical ventilation by a computer-controlled piston. Pressure-volume data were captured using a step-wise, quasi-static inflation/deflation maneuver using a PEEP of 3 cmH_2_O and tidal volume of 10 mL/kg.

Functional cardiac testing was done by echocardiography using the Vevo2100 high-frequency imaging system (Visualsonics, SonoSite, Inc., Toronto, Ontario) under light isoflurane anesthesia as previously described [27]. The Vevo2100 M700 (50 MHz) transducer was used for neonates and the M250 (21MHz) transducer was used for older rats. Cardiac images were captured in B-Mode (brightness mode), M-Mode (motion mode) and Pulsed-Wave Doppler Mode (PW) using parasternal long axis (PLAX), parasternal short axis (PSAX), and the apical four chamber views. After noticing a high rate of cyanotic and dying newborns in the HF diet-exposed groups, detailed pulmonary artery views were obtained in a subset of each group. Image analysis was done using the Vevo2100 Imaging System Software. Because right ventricular (RV) shape is subjective and tricuspid regurgitant jet is not a sensitive marker for PPHN, we selected pulmonary artery (PA) flow parameters as the best non-invasive, quantifiable measures in our model. Although mean and peak PA gradients and velocities can be indicative of elevated pulmonary pressures, the use of PA acceleration time (PAAT) provides an accurate estimate of the peak systolic PA pressure independent of tricuspid regurgitation [31–33]. The RV ejection time (RVET) was also measured to allow an adjustment for heart rate through the calculation of the PAAT/RVET ratio which serves a quantifiable and non-invasive measure of mean PA pressures [32].

### Molecular Analyses

As previously described [34], snap frozen lungs were lysed in 50mM Tris (pH 7.4), 150mM NaCl, 0.2% Triton X-100, 0.3% NP-40 and 0.1mM phenylmethylsulfonyl fluoride (PMSF) supplemented with protease and phosphatase inhibitor cocktails (1:1000, Sigma-Aldrich). Protein concentrations were determined by a Modified Pierce BCA Protein Assay (Thermo Scientific). Cell lysates were analyzed by Western blot and Milliplex assays. For blots, lung lysate was diluted with 3X Laemmli buffer, separated by SDS-PAGE, and transferred to polyvinylidene difluoride membranes. Membranes were incubated with anti-thioredoxin-interacting protein or Txnip (1:500, MBL International, Woburn, MA), anti-T1α (1:1000, Epitomics, Cambridge, MA), anti-surfactant protein B or SP-B (1:1000, Chemicon), anti-surfactant protein C or SP-C (1:1000, Santa Cruz, Dallas, TX) and β-actin (1:1000, Sigma-Aldrich) antibodies in 5% nonfat milk. Membranes were then incubated at room temperature with anti-rabbit (1:5000, Southern Biotechnology) or anti-mouse (1:5000, Southern Biotechnology) HRP-conjugated secondary antibody in TBS-T with 5% nonfat milk and visualized by chemiluminescence using a UVP Biospectrum 500 imaging system (UVP, Upland, CA). Densitometry was performed on blots using the UVP Visionworks LS Software.

Milliplex assays performed on lung lysate were normalized to protein concentration and repeated in duplicate or triplicate. Samples were assayed using industry provided standards and analyzed with the Luminex 200 Milliplex Analyzer as described by the manufacturer. Milliplex multi-analyte panels for total and phosphorylated (activated) protein kinase B (Akt) and mitogen-activated protein kinase or MAPK (ERK1 and 2) were run on a single plate using 25 μg of lung lysate protein per well. Results were reported qualitatively in median fluorescence intensity units (MFI). The activated:total ratio was calculated for individual samples and then averaged per group. Endothelin-1 (ET-1) was assayed on lung protein (350 μg of protein/sample) using the ET-1 rat ELISA kit (Enzo Life Sciences, Farmingdale NY) according to the manufacturer’s instructions. An ET-1 extraction for samples with low levels of ET-1 was performed utilizing Sep-Pak columns according to the kit’s manufacturer’s instructions prior to the assay. Results are reported in pg/mg of lung protein. Lastly, vascular endothelial growth factor-A (VEGF) was quantitatively assayed on newborn lung lysate (222 μg of protein/well) by Milliplex assay; results are reported in pg/μg of protein.

### Statistical Analyses

Descriptive data are expressed as means ± SEM unless otherwise noted. Data was interrogated using GraphPad Prism 5 (Lajolla, CA). At first, datasets were analyzed by two-way ANOVA with a Bonferroni post-test to interrogate diet, diabetes and interaction effects. The final model reports diet and diabetes-related effects, but these were only considered if the interaction term was not significant at 5% level. When interaction was significant, further clarification was required, and differences between controls and each exposed group were determined using one-way ANOVA with Dunnett’s multiple comparison post-hoc analysis. Linear regression was used to compare maternal data over time (weight, serum TG). Perinatal mortality was compared using logistic regression (Proc Genmod in SAS 9.3) with a repeated statement to account for clustering of pups within moms. Interaction of drug and diet was removed from the mortality model since it was not significant. For all analyses, the level of significance was set at p≤0.05.

## Results

### Model characteristics

Dams and offspring from 48 litters (controls, n = 12; diabetes-exposed, n = 13; HF diet-exposed, n = 12; combination-exposed, n = 11) with 486 live born pups were used to characterize the features of our animal model. All live offspring were weighed and had echocardiography. Thereafter, pups were stratified for evaluation of both cardiac and pulmonary outcomes at either newborn or 3 week time-points. Model characteristics and cardiac outcomes have been previously described in detail for these litters [27] and are summarized in S1 Table. In short, dams on a HF-diet had greater weight gain over time and higher circulating leptin levels. Dams with late-gestation diabetes had significantly higher blood glucose levels after GD14 administration of STZ (between 310–360 mg/dl vs. 90-95mg/dl on average, p≤0.001). Both diabetes (p = 0.04) and HF diet (p = 0.02) were associated with higher circulating maternal TG levels so that just after delivery, diabetes-exposed dams had a 2-fold higher serum TG, HF diet-exposed dams had a 3-fold higher serum TG, and combination-exposed dams had a 15-fold higher serum TG (mean = 555mg/dl) compared to controls. Both diabetes-exposed (p = 0.002) and HF diet-exposed (p = 0.009) newborn offspring had higher serum insulin levels leading to a 10-fold higher average circulating insulin level in combination-exposed pups compared to controls. Serum TG levels were not different between offspring groups [27]. Cytokines, measured in a subset of 13–26 newborn pups/litter (S1 Table), demonstrated higher circulating interleukin-6 (IL-6) levels in combination-exposed offspring (p = 0.006). A trend towards higher circulating tumor necrosis factor α (TNFα) was also found in combination-exposed pups (p = 0.08).

### Offspring Mortality

**Fig 2:**
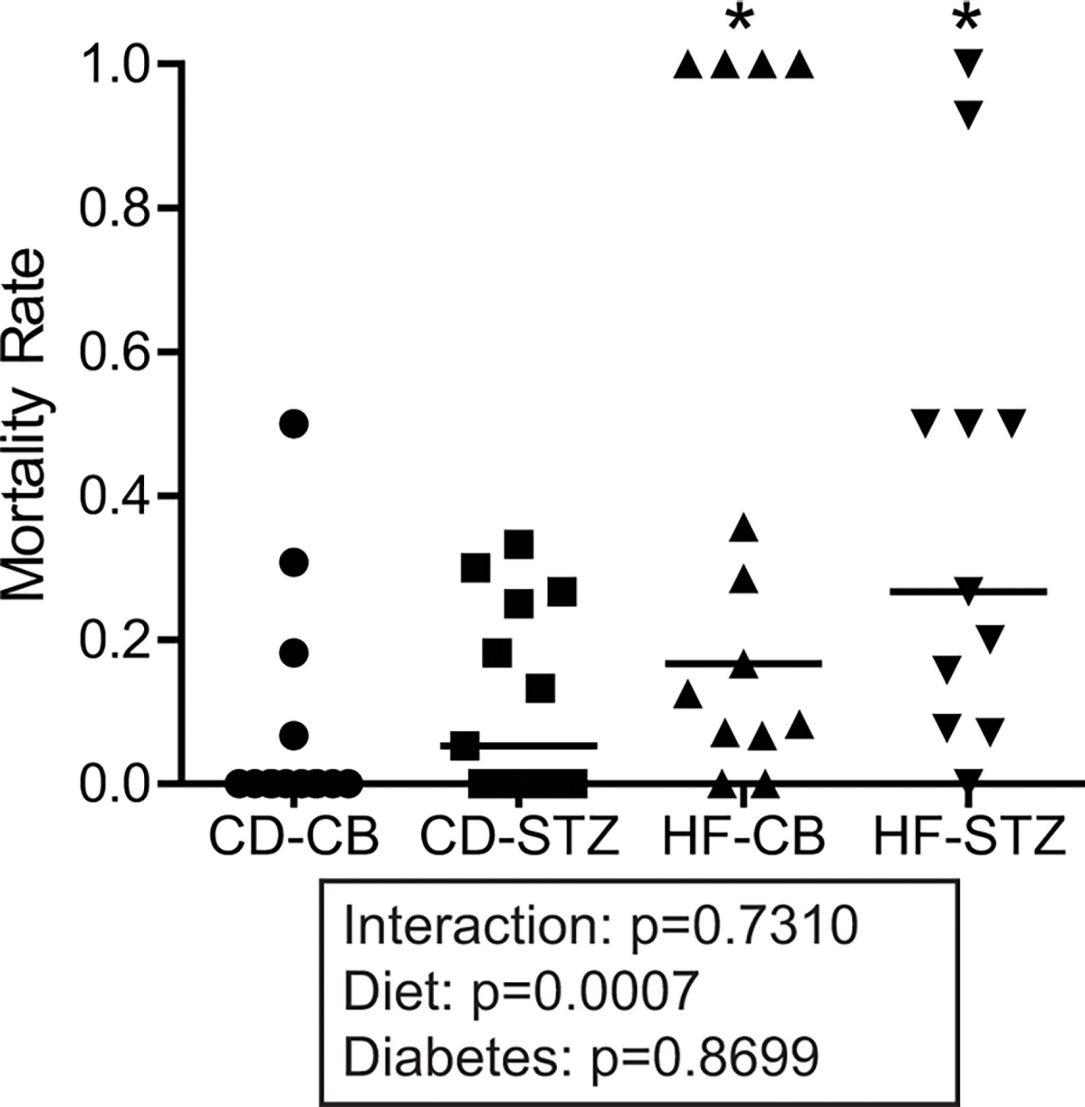
Perinatal mortality rate. Perinatal mortality is expressed by group as a scatterplot. Individual marks represent the percent of each litter that is dead by newborn day one. The average mortality rate is represented by the median (horizontal bar). * indicates diet associated differences by 2-way ANOVA (p<0.05). CD-CB, controls; CD-STZ, diabetes exposed; HF-CB, high-fat diet exposed; HF-STZ, combination (high-fat and diabetes) exposed. N = 11–13 litters per group.

One unanticipated observation of the study was a significantly higher perinatal mortality rate in HF diet-exposed (p = 0.0007), but not diabetes-exposed (p = 0.87) offspring (Fig 2). The odds of perinatal death being associated with exposure to a maternal HF diet were 89% higher than exposure to a CD. Mortality rates included stillbirths (pups found dead or resorbing *in utero*), pups found dead in the cage and those that died or were dying during observation on the day of delivery (Table 1). Pups that were found alive but were dying on the first day of life were often observed to have respiratory distress, cyanosis, and inability to feed. Combination-exposed pups that survived to cross-fostering had the highest spontaneous death rate between newborn and later endpoints (Table 1).

**Table 1:** Offspring mortality time points. Data are expressed as sums (percent of total pups) per group that were found alive, dead in the cage, dying or dead in utero on the day of delivery.

Group	Litters per group	Total placentations per group	Live on day 1	Found dead in the cage on day 1	Dying on day 1	Found dead *in utero* (stillbirth)	*Died after cross-fostering on day 1
Controls	12	128	117 (91%)	11 (9%)	8 (6%)	3 (2%)	0 (0%)
Diabetes exposed	13	141	126 (89%)	15 (11%)	9 (6%)	6 (4%)	2 (1%)
Diet exposed	12	120	80 (67%)	40 (33%)	14 (12%)	26 (22%)	4 (3%)
Combination exposed	11	122	82 (67%)	40 (33%)	26 (21%)	14 (11%)	7 (6%)

* The last column is the sum (percent) of total pups per group that died naturally after cross-fostering on the first day of life.

### Offspring Morphometry

Morphometric data are presented in Table 2. Despite finding no significant difference in the average litter size (controls 10.7, diabetes-exposed 10.9, diet-exposed 10.73, combination-exposed 11.1, p>0.05), birth weight was lower in the combination-exposed offspring compared to controls. Weight remained significantly lower in this group at 3 weeks of age. A significant interaction effect was found (p = 0.0003) at that time point when the HF diet-exposed pups were heavier than controls, but combination exposed pups continued to lag in weight gain. Diabetes-exposed, diet-exposed and combination-exposed offspring had larger hearts and smaller lungs at birth. By 3 weeks, the difference in lung size was no longer present. However, combination-exposed offspring continued to have a greater heart:body weight ratio.

**Table 2:** Offspring morphometry. Data collected at various newborn (NB1) and 3-week-old (weaning) time-points are expressed as mean ± SEM. Arrows indicate significant differences related to * diet or ^±^ diabetes by 2-way ANOVA.

Group	Age	Weight (gm)	Heart:body weight (ratio)	Lung:body weight (ratio)
**Controls**	NB1	6.05±0.10, n = 125	0.0071±0.0002, n = 68	0.029±0.0016, n = 14
**Diabetes exposed**	NB1	5.81±0.09, n = 130	^**±**^**0.0077±0.0002,** n = 77 **↑**	^**†**^**0.025±0.0009,** n = 28**↓**
**Diet exposed**	NB1	5.72±0.10, n = 112	*0.0089±0.0003, n = 89 **↑**	^**†**^**0.021±0.0010,** n = 6 **↓**
**Combination exposed**	NB1	^**†**^**5.77±0.13,** n = 119**↓**	^±^*0.0094±0.0003, n = 66**↑**	^**†**^**0.023±0.0007,** n = 14**↓**
**Controls**	3 week	58.58±2.22, n = 20	0.0070±0.0003, n = 19	0.0097±0.0002, n = 3
**Diabetes exposed**	3 week	62.25±2.87, n = 16	0.0068±0.0003, n = 14	0.0095±0.0006, n = 6
**Diet exposed**	3 week	65.76±1.90, n = 14	0.0065±0.0002, n = 11	0.0087±0.0004, n = 3
**Combination exposed**	3 week	^**†**^**47.09±3.74,** n = 15**↓**	^**†**^**0.0080±0.0006,** n = 15	0.0097±0.0005, n = 6

^†^ Interaction effect was found and significant changes indicated are by one-way ANOVA with Dunnett’s post-test comparing to controls. Significance set at p<0.05.

### Lung Histology

#### Alveoli and Vessel Histology in Newborn Offspring

**Fig 3:**
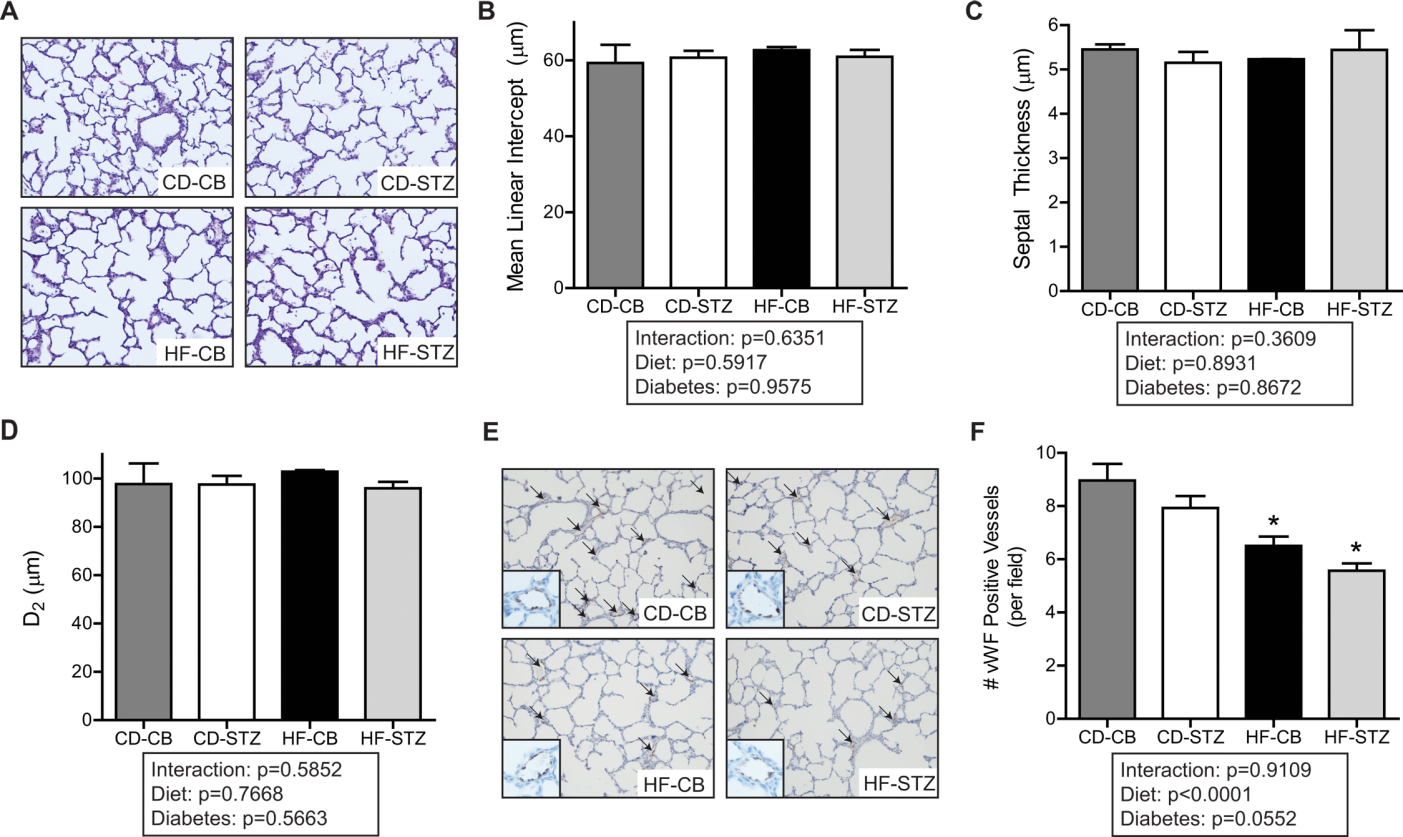
Alveolar and vessel histology in newborn lungs. (A) Representative hematoxylin and eosin staining in fixed newborn rat lung (20X). Comparison of (B) mean linear intercept, (C) septal thickness, and (D) airspace area (D_2_) as quantified using ImageJ to analyze five images per lung with four animals per litter (n = 2–4 litters/group). (E) Representative immunohistochemistry of fixed newborn rat lung processed with von Willebrand factor (vWF) stain to identify endothelial cells (indicated by arrows; see inset for staining details). (F) vWF-positive vessels were quantified from five representative images per lung with four animals per litter (n = 2–4 litters/group). Data are expressed as mean±SEM across treatment groups. * indicates significant diet associated changes by 2-way ANOVA. Significance set at p<0.05. CD-CB, controls; CD-STZ, diabetes exposed; HF-CB, high-fat diet exposed; HF-STZ, combination (high-fat and diabetes) exposed.

Alveolarization, as measured by mean linear intercept, septal thickness, and airspace area (D2) were not apparently different among groups at birth (Fig 3). Alveolar proliferation, as assessed by Ki67 staining, was robust at newborn but not 3 week time-points and was not qualitatively different between exposure groups. Apoptosis, as assessed by TUNEL staining, was nearly absent at either time-point and was not different between groups. The most obvious histologic finding was fewer pulmonary vessels (positive for von Willebrand Factor) demonstrated and enumerated in HF-diet exposed newborn pups, regardless of maternal diabetic status (Fig 3E and 3F).

#### Markers of Lung Maturity in Newborn Offspring

**Fig 4:**
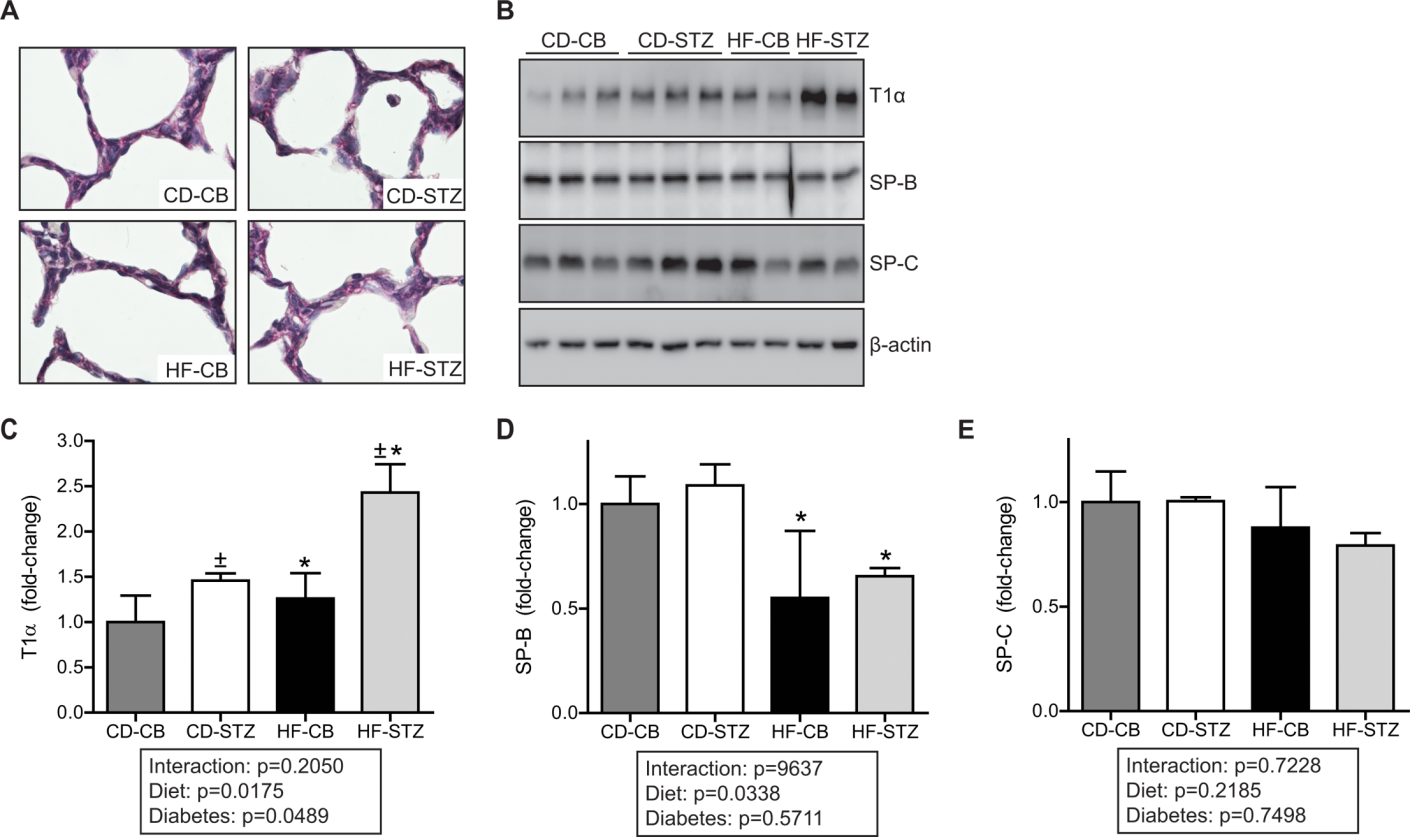
Maturity assessment in newborn lungs. (A) Representative glycogen deposition in fixed newborn rat lung stained using PAS stain (60X). (B) SDS-PAGE and immunoblot of newborn rat lung lysates using anti-T1α, anti-SP-B and anti-SP-C with β-actin as a loading control. Each lane represents equal protein contributions of multiple offspring (n = 2–4) from a single litter. Densitometric analysis of (C) T1α (D) SP-B and (E) SP-C protein expression. Data are expressed as mean±SEM. * indicates significant diet and ^±^ diabetes associated changes by 2-way ANOVA. Significance set at p<0.05. CD-CB, controls; CD-STZ, diabetes exposed; HF-CB, high-fat diet exposed; HF-STZ, combination (high-fat and diabetes) exposed.

Lung maturity was assessed by PAS staining, T1α protein expression as a marker of Type I cell content, and surfactant protein (B and C) expression (as a marker of Type II cell content/function). Results are shown in Fig 4. Glycogen stores are stained red by PAS as shown in Fig 4A and were not apparently different between exposed offspring groups at either newborn or three week time points. Lung protein expression of T1α was higher in both diabetes-exposed and HF diet-exposed offspring (Fig 4B and 4C). Expression of SP-B was lower in HF diet-exposed newborns pups, and expression of SP-C trended lower in all exposed pups but did not reach statistical significance (Fig 4B, 4D and 4E).

#### Alveoli and Vessel Histology in 3-Week-Old Offspring

**Fig 5:**
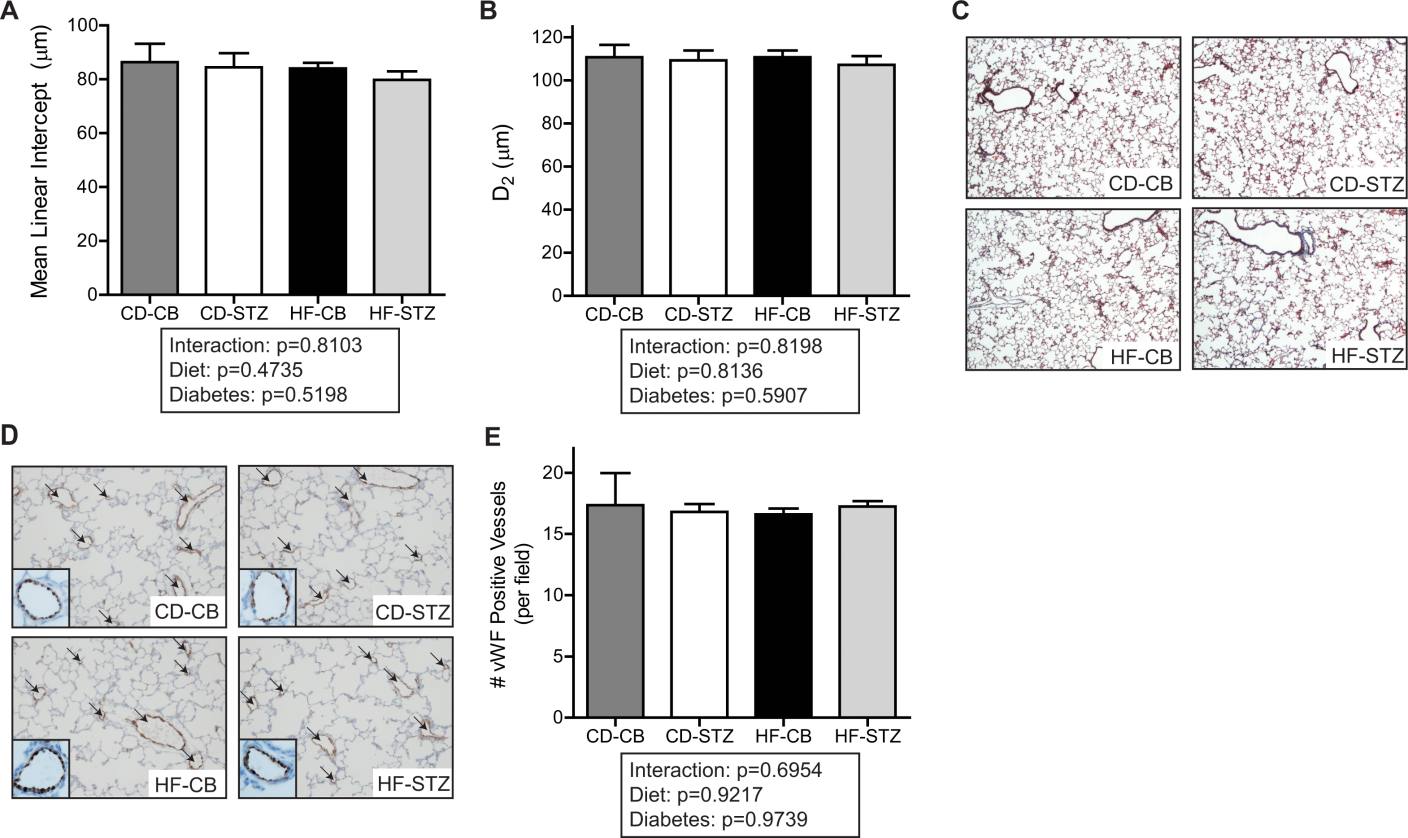
Alveolar and vessel histology in 3-week-old lungs. Quantification of (A) mean linear intercept and (B) airspace area (D_2_) using ImageJ to analyze five images per lung across four animals per litter (n = 4 litters). Representative images of 3 week-old rat lungs stained to identify (C) collagen deposition with trichrome staining (10X) and (D) endothelial cells using immunohistochemistry for von Willebrand factor (vWF) (endothelial cells are indicated by arrows; see inset for details). (E) vWF-positive vessels were quantified from five representative images per lung with four animals per litter (n = 4 litters). Data are expressed as mean±SEM across treatment groups. CD-CB, controls; CD-STZ, diabetes exposed; HF-CB, high-fat diet exposed; HF-STZ, combination (high-fat and diabetes) exposed.

Alveolarization, as measured by mean linear intercept and airspace area (D2) remained similar among groups at 3 weeks of age (Fig 5A–5C). There was difference in septal thickening. As mentioned above, Ki67 staining for proliferation diminished from the newborn to 3 week time-point in all groups similarly. TUNEL and PAS staining were also not different between groups. Trichrome staining was evaluated as a marker of fibrosis at this later developmental time-point only, but no qualitative difference was found among exposed offspring. Although diet-exposed pups had fewer pulmonary vessels (positive for von Willebrand Factor) enumerated at the newborn time-point, this group difference was no longer found by 3 weeks of age. Of note, analyses at this time-point cannot account for the pups that died after cross-fostering, which was also higher in the HF diet-exposed groups.

### Physiology

#### Hemodynamics

Although a high perinatal death rate in diet-exposed pups did not allow us to capture echocardiography in the most severely affected offspring, surviving newborns exposed to a maternal HF diet demonstrated echocardiographic evidence of PPHN (Table 3). These diet-exposed pups were often observed to have a D-shaped right ventricle (RV) and/or tricuspid regurgitation on echocardiography. PAAT, RVET and PAAT/RVET ratio (which serve as a more objective marker of PPHN) were analyzed in a subset of newborn pups for comparison. PAAT, an estimate of the peak systolic PA pressure independent of tricuspid regurgitation [32], was significantly decreased in HF diet-exposed offspring (p = 0.02). The PAAT/RVET ratio, which adjusts for heart rate differences between animals, was also significantly lower (p = 0.03) in diet-exposed pups. At 3 weeks of age, surviving offspring diabetes-exposed, but not diet-exposed, had a lower peak PA gradient and velocity. And although no significant interaction effect was found, combination-exposed offspring had the lowest peak and mean PA gradient, lowest velocity and PAAT/RVET, and the highest mortality rate after the newborn period.

**Table 3:** Offspring echocardiography.

Group	Age	PA peak gradient(mmHg)	PA peak velocity (mm/s)	PAAT(msec)	RVET(msec)	PAAT/RVET(ratio)
**Controls** n = 94/6	NB1	0.84±0.04	448±10	27.83±2.52	100±2.70	0.28±0.026
**Diabetes exposed** n = 98/12	NB1	0.78±0.04	428±11	28.42±3.89	111±2.98	0.25±0.030
**Diet exposed** n = 80/8	NB1	^†^**0.70±0.04↓**	^†^**403±13↓**	*20.63±0.99↓	100±4.19	*0.21±0.015↓
**Combination exposed** n = 51/6	NB1	0.81±0.05	440±16	*19.17±2.33↓	97.8±5.60	*0.19±0.016↓
**Controls** n = 15	3weeks	2.09±0.23	710±37	21.41±1.11	87.70±4.55	0.25±0.02
**Diabetes exposed** n = 14	3weeks	^**±**^**1.57±0.16↓**	^**±**^**618±30↓**	21.98±1.90	91.07±4.89	0.24±0.01
**Diet exposed** n = 17	3weeks	1.76±0.14	656±24	22.55±1.50	82.81±2.43	0.27±0.01
**Combination exposed** n = 14	3weeks	^**±**^**1.35±0.15↓**	^**±**^**569±32↓**	21.35±1.37	89.35±3.57	0.23±0.01

Data collected at various time-points are expressed as mean±SEM. Arrows indicate significant differences (p<0.05) related to *diet or ^±^diabetes by 2-way ANOVA.

^†^Interaction effect was found and significant changes that are indicated remained after query by one-way ANOVA with Dunnett’s post-test comparing to controls. PA, pulmonary artery; PAAT, PA acceleration time, (an estimate of the peak systolic PA pressure independent of tricuspid regurgitation); RVET, right ventricular ejection time.

#### Pulmonary Function in 3-Week-Old Offspring

**Fig 6:**
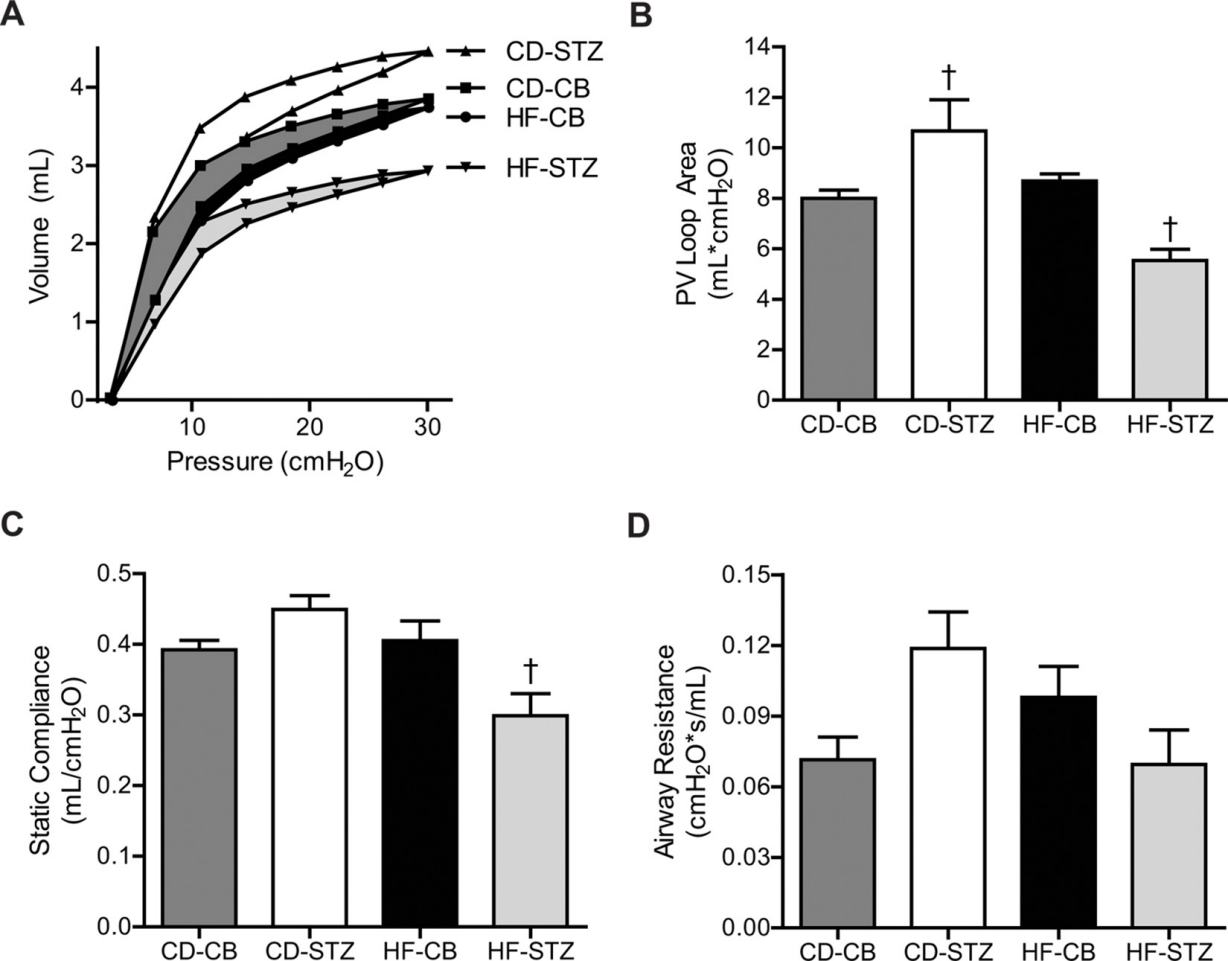
Pulmonary function testing in 3-week-old offspring. Respiratory physiology was evaluated in 3-week-old offspring via mechanical ventilation using the FlexiVent FX system. Computer-controlled perturbations measured (A) pressure-volume curves, (B) PV loop area (C) static compliance and (D) airway resistance. Quantifiable data are expressed as mean±SEM (n = 3–7 per group). A significant interaction effect was found in all measures by two-way ANOVA and differences that remained significant by one-way ANOVA with Dunnett’s post-test comparison to controls are indicated by ^†^. Significance set at p<0.05. CD-CB, controls; CD-STZ, diabetes exposed; HF-CB, high-fat diet exposed; HF-STZ, combination (high-fat and diabetes) exposed.

Pulmonary function testing, as a more sensitive marker of lung disease over time, was analyzed in surviving 3-week-old offspring from each group. Combination-exposed offspring had a lower maximal lung volume capacity and a flatter hysteresis curve suggestive of poor compliance or restrictive lung disease (Fig 6A). A significant interaction effect was found when comparing group differences in specific pulmonary physiologic markers. Interestingly, diabetes only-exposed offspring demonstrated the most compliant lungs with a significantly higher pressure-volume loop area (Fig 6A and 6B). However, combination-exposed offspring had the least compliant lungs with a significantly lower pressure-volume loop area and reduced static compliance (Fig 6A–6C). Although there was also an interaction effect found when comparing airway resistance, no differences remained by Dunnett post-test analyses comparing exposed groups to controls (Fig 6D).

### Mechanistic Inquiry

Given our findings, plausible pathogenic mechanisms were explored and are represented in Table 4. It is known that hyperglycemic, hyperlipidemic and hyperinsulinemic conditions alter activation of the Akt and MAPK pathways, which are upstream regulators of cellular proliferation and vascular reactivity. At the newborn time point, lung lysate from diabetes-exposed (p = 0.025), but not diet-exposed (p = 0.90), offspring had lower expression of activated pAkt, an upstream activator of endothelial derived nitric oxide (NO). Total lung Akt expression was higher in the HF diet-exposed pups, yielding an overall lower pAkt:Akt ratio. Within the parallel MAPK/ET-1 pathway, diabetes-exposed (p = 0.016), but not diet-exposed (p = 0.24), pups had a lower expression of both total and phosphorylated (activated) ERK, yielding no difference in the pERK/ERK ratio between newborn groups. However, expression of downstream ET-1, an endothelial derived vasoconstrictor, trended higher in all exposed offspring and was significantly higher in combination-exposed offspring. Serum VEGF levels were not different between groups. However, lung VEGF levels were significantly lower in both diabetes-exposed and diet-exposed offspring compared to controls (Table 4). Additionally, HF diet-exposed offspring have significantly lower levels of Txnip, the redox-sensitive scaffolding protein which is necessary for sustained VEGF mediated signaling and new vessel formation [35].

**Table 4:** Akt, MAPK and VEGF expression in newborn lung lysates.

Group	pAkt (MFI)	Total Akt (MFI)	pAkt:Akt (ratio)	pERK (MFI)	Total ERK (MFI)	pERK:ERK (ratio)	ET-1 (pg/mg)	VEGF (pg/μg)
**Controls** n = 8–11	31.5±2.4	289±84	0.25±0.06	65.9±9.7	3792±346	0.017±0.001	18.7±4.0	0.16±0.02
**Diabetes exposed** n = 9–11	^**±**^**25.6±1.9↓**	190±56	0.26±0.05	^**±**^**45.9±5.0↓**	^**±**^**2811±282↓**	0.016±0.001	26.8±2.2	^**†**^**0.07±0.02↓**
**Diet exposed** n = 7–11	30.9±2.7	^*†^**1214±333↑**	^**†**^**0.14±0.05↓**	54.4±3.9	3775±229	0.015±0.001	^*^36.8±5.5↑	^**†**^**0.04±0.01↓**
**Combination exposed** n = 9–11	^**±**^**25.6±2.5↓**	313±132	0.25±0.07	^**±**^**42.3±5.3↓**	^**±**^**3095±354↓**	0.014±0.001	28.9±3.0	0.11±0.02

Total and activated (phosphorylated) Akt and MAPK (ERK1/2) expression from newborn lung lysate (25 μg of protein per well) were assayed using a Milliplex multi-analyte panels and are reported qualitatively in median fluorescence intensity units (MFI). ET-1 as measured by ELISA and VEGF-A as measured by Milliplex assay are reported as pg/mg of lung protein. Data are expressed as mean±SEM. Arrows indicate significant differences (p<0.05) related to *diet or ^±^diabetes by 2-way ANOVA. When an interaction effect was found, the differences that remained significant by one-way ANOVA with Dunnett’s post-test comparison to controls are indicated by ^†^. Akt, protein kinase B; mitogen-activated protein kinases, includes ERK1 and ERK2; vascular endothelial growth factor-A, VEGF; MFI, median fluorescence intensity units in 25μg of protein; ET-1, endothelin 1; p, phosphorylated (activated).

## Discussion

This study sheds important light on the effects of a maternal HF diet, alone and alongside late-gestation diabetes, on alveologenesis and vasculogenesis in the developing fetal lung. Of most interest, our rat model demonstrates that offspring exposed to a maternal HF diet, but not diabetes, have fewer pulmonary vessels, PPHN and a higher mortality rate. Moreover, the combination of maternal HF diet and late-gestation diabetes negatively impacts fetal lung maturity and pulmonary function at 3 weeks of age. After probing various plausible mechanisms of pathogenesis, we found that these consequences likely stem from multiple pathways. Similar studies of this nature are limited, and evidence gained helps answer four understudied questions about fuel-mediated effects on fetal lung development.

### Novel Questions Answered

#### Maternal diabetes that begins after primary organogenesis does not alter structural lung development

Prior studies demonstrating negative effects of maternal diabetes on fetal lung development used lung explants exposed to very high concentrations of glucose [10] or induced hyperglycemia before primary organogenesis was complete (before breeding or shortly after mating) [10, 15, 16, 36]. We found that when maternal diabetes was induced late in pregnancy (GD14 of 22), no significant *structural* alveolar differences were found. This is important because the most common type of diabetic pregnancy is gestational diabetes, which is diagnosed and treated late in pregnancy. However, caution should be taken in translating our rat model findings to women with gestational diabetes because STZ-induced diabetes is quite different, and women with gestational diabetes have a high rate of co-morbid obesity. The normal rat diet (CD) is low in fat, but the typical Westernized diet is more similar to the HF diet used in our study. Although diabetes-associated *structural* lung changes were not found, late-gestation diabetes alongside a maternal HF diet was associated with delayed pulmonary maturation (lower surfactant protein B) and poorer lung compliance in offspring at 3 weeks of age. It should also be noted that we did not do a bronchoalvealar lavage to further investigate surfactant quantity or quality. Therefore, further clarification about the effects of *late*-gestation diabetes on fetal pulmonary maturation is warranted.

#### A maternal HF diet, alone and alongside late-gestation diabetes, increases pulmonary morbidity in the developing offspring

One previous study by Mayor et al. reported the effects of a maternal HF diet (42% calories from fat) on fetal lung development [24]. Like us, they found a significantly higher perinatal death rate and lower fetal weight (E18). Mayor et al. did not find differences in alveolar chord lengths, but they reported delayed fetal lung maturation and a lower mean linear intercept in HF diet-exposed offspring at postnatal day 15 [24]. This measure, which accounts for both alveolar and ductal space, is dependent on lung volume. Mayor’s finding is puzzling, because simplified/immature lungs with diminished proliferation would be expected to have less alveolar septations/more air space and thus a higher mean linear intercept [37]. Perhaps total lung volume could account for their finding, as they report a reduced fetal (E18) size without a reduced lung weight. Using our model, we did not find diet-related differences in alveolarization at earlier or later developmental time-points (NB1 and 3 weeks vs. P14). Offspring in Mayor’s study were not cross-fostered to normal dams, so postnatal influences (feeding from HF-fed mothers) could account for differences in the mean linear intercept found on postnatal day 14.

In regards to lung maturation, Mayor reported a 35% reduction in proliferation (Ki67 staining), higher glycogen content (PAS staining) and lower surfactant (B, C and D) RNA expression [24]. Although we did not see striking differences in proliferation (by Ki67), apoptosis (TUNEL staining) or PAS staining at newborn or 3-week time points, we did find some evidence of a HF diet-induced delay in pulmonary maturation. T1α expression, a marker of Type I pneumocytes, was significantly higher in both diabetes-exposed and HF diet-exposed newborn lungs. Like Mayor, newborn lungs exposed to a maternal HF diet in our study had lower surfactant protein B (and potentially C) protein expression. Therefore, both studies show that a maternal HF diet during pregnancy is associated with delayed fetal lung maturation. Perhaps the most striking finding from our study is that a maternal HF diet was also independently associated with fewer pulmonary endothelial cells (positive for von Willebrand Factor), which brings us to the most interesting finding.

#### A maternal HF diet influences fetal pulmonary vascular development

To our knowledge, we are the first to show that a maternal HF diet negatively affects pulmonary vascular development and cardiopulmonary hemodynamics and increases perinatal mortality in newborn offspring. Despite the known risk of PPHN in infants born to obese or diabetic mothers [8, 17], very few studies have investigated the effect of excess circulating fuels on pulmonary vascular development. Sosenko et al. reported a decrease in alveolar capillary density in diabetes-exposed rabbit offspring, but Koskinin et al. found no significant differences in pulmonary vascular organization by micro-CT in diabetes-exposed mice on postnatal day 14 [15]. Fan et al. showed evidence of *systemic* endothelial dysfunction in juvenile non-human primates exposed to a maternal HF diet (impaired vasorelaxation, intimal thickening of the abdominal aorta and elevated levels of vascular inflammatory markers), but pulmonary vessels were not evaluated, and these offspring were also breastfed by HF-fed mothers [38]. Our study is the first to investigate the pulmonary vascular consequences of a maternal HF diet during pregnancy. In doing so, we found that HF diet-exposed, but not diabetes-exposed, offspring had fewer pulmonary vessels enumerated and echocardiographic evidence of PPHN. Given that there was no synergistic effect found in combination-exposed pups, we surmise that this effect is directly related to dietary fat intake. This is a significant finding that should guide future studies to examine a potential correlation between maternal dyslipidemia and PPHN.

#### Prenatal exposure to a maternal high-fat diet and diabetes is associated with pulmonary morbidity beyond the perinatal period

This study highlights that prenatal exposure to a maternal HF diet and diabetes is sufficient to cause pulmonary consequences beyond the perinatal period, even when postnatal influences are equalized. A significant strength of our study is that we equalized litter size and cross-fostered pups to normal dams to limit confounding from postnatal exposure and followed outcomes over time. Additionally, offspring were evaluated using pulmonary function testing which may be a more sensitive marker of developmental lung disease than histology alone. In doing this, we found that combination-exposed offspring had poorer lung compliance at 3 weeks of age. The underlying pathogenesis is still unknown but likely stems from various causes. Postnatal lung development is dependent on a normal ratio of Type I and Type II cells (37). We found a higher expression of T1α, a marker of Type I cells, in the both diabetes-exposed and HF diet-exposed newborn lungs. We also found a lower expression of surfactant protein B, a marker of Type II cells in HF diet-exposed lungs. This newborn finding could contribute to altered postnatal lung development and poorer pulmonary function at weaning. Additionally, vascular signaling [21] is important in postnatal lung development, so alterations in pulmonary vasculogenesis associated with a maternal HF diet could also play a role in pathogenesis. Mayor et al. also hypothesized that HF-diet associated delays in fetal lung maturation was instigated by placental inflammation and glucocorticoid receptor alterations [24]. Our findings add credibility to Mayor’s hypothesis. In our study, combination-exposed offspring were subjected to the most severe maternal dyslipidemia, had the highest levels of circulating cytokine levels at birth and had the most striking decline in pulmonary compliance at 3 weeks. In fact, diabetes-exposed offspring in our study had the lowest circulating cytokine levels at birth and better lung function than controls at 3 weeks. For these reasons, we do not think that hyperglycemia or insulin exposure alone is associated with the same lasting effects. We agree that influences of inflammation on the developing lung, such as increased surfactant turnover, oxidative injury or fibrosis, may play a role. Although we did not see a qualitative difference in fibrosis by Trichrome staining at 3 weeks of age, more detailed investigation is warranted and should include additional later time-points.

### Proposed Mechanisms of Pathogenesis

Combined, these studies establish that a maternal HF diet is associated with detrimental pulmonary consequences for the developing fetus, especially when combined with late gestation diabetes. How this occurs may be answered in part by Mayor’s inflammatory hypothesis and by additional mechanisms of pathogenesis. Glucose, insulin, fatty acids and cytokines are all modifiers of the Akt/NO and VEGF production pathways [39] that are essential for normal alveolar maturation [40] and vascular growth and reactivity [41]. Likewise, the insulin-regulated MAPK pathway is crucial for lung development [42] and acts upstream of ET-1, which plays an important role in pulmonary vascular resistance [43]. Unfortunately, vessels exposed to hyperinsulinemia often develop a selective impairment of Akt/NO pathway activation over time, whereas the MAPK/ET-1 dependent pathway often remains unaffected [44, 45], leading to an imbalance that favors vasoconstriction. Using our rat model, we found a fuel-mediated decrease in net AKT activation (upstream of NO production) alongside higher ET-1 production that could contribute to maladaptation of the pulmonary vasculature bed in the transitioning newborn lung. Further investigation of eNOS phosphorylation, NO and ET-1 antagonist rescue should be considered in the future. Congruently, diabetes-exposed and HF diet-exposed offspring had lower lung expression of VEGF and its redox-sensitive scaffolding protein Txnip, which is necessary for sustained signaling for endothelial cell proliferation, migration, tube formation and vessel growth [35, 46]. Interestingly, previous studies have demonstrated late restrictive lung disease with VEGF inhibition [47]. In sum, our findings suggest multiple mechanisms that could lead to pulmonary vascular maladaptation and maldevelopment leading to PPHN at birth and functional lung disease over time in exposed offspring.

### Study Limitations

#### Model Limitations

Our rat model was designed to investigate the effects of a maternal HF diet and late-gestation (rather than pre-gestational) diabetes on fetal lung development. Rat diets were selected to translate to commonly attainable low fat or HF dietary “lifestyle” [48] and were not intended to be obesogenic. Therefore, maternal obesity may not impart the same consequences. Although STZ-induced pancreatic beta cell injury does not incite immune-mediated Type 1 diabetes or insulin-resistant Type 2 diabetes, our model yields the desired triad of maternal hyperglycemia, hyperlipidemia, and fetal hyperinsulinemia necessary to study the individual and combined effects, irrespective of the underlying cause. Diabetes was not induced until GD14 to eliminate the confounding effects of diabetes on placentation (early loss) and early organogenesis. Also, dams were partially treated with insulin to mimic treatment of diabetes after a screening glucose tolerance test at 24–28 weeks gestation in pregnant women. Our study investigates only prenatal effects, because after birth, litters were culled to equal size, and offspring were cross-fostered to normal mothers to mature without the confounding effects from postnatal nutrition and rearing.

#### Limitations for Pulmonary Alveologenesis Interpretation

One notable caveat is that when interpreting data from a rat model, one must consider the relatively delayed lung maturation compared to humans. Rats typically deliver when lungs are in the saccular phase, and alveolar development does not usually occur until after birth [6]. Additionally, mean linear intercept, septal thickness, and airspace area (D2) are typically used to quantify alveologenesis, but investigation of lung maturity was limited. Bronchoalveolar lavage was not performed prior to inflation fixation and so qualitative surfactant analyses (airspace protein and lipid content) could not be performed, leading to a lack of biochemical data that could clarify fuel-mediated effects on fetal lung maturity. In addition, although pulmonary function testing may be a more sensitive marker of pulmonary morbidity than histology, an unexpectedly high mortality rate found in the HF diet-exposed offspring confounds our physiologic data, because only live offspring (likely the healthiest of the litters) could be tested. Additionally, both Mayor’s study [24] and our study demonstrate that rodents exposed to a maternal HF diet are growth-restricted. We attempted to correct for small size by comparing the lung:body weight ratio as a relative marker of lung growth, and at the time that pulmonary function testing was done, this was not different between groups. However, it is certainly plausible that intrauterine growth restriction itself is associated with altered lung development [49] or delayed pulmonary maturation [50] as previously reported by others.

#### Limitations for Pulmonary Vasculogenesis Interpretation

Although fewer pulmonary vessels (positive for von Willebrand Factor) were demonstrated and enumerated, endothelial cell proliferation, apoptosis and arterial remodeling were not specifically analyzed. Pulmonary circulatory imaging (micro-CT) was not available and echocardiographic evidence of PPHN was also confounded by a high mortality rate in HF-diet exposed offspring. Although never noticed during echocardiography, we did not document evidence of a closed ductus arteriosus, which can account for pulmonary over circulation and a high mortality rate in the first day of life in rodents [51]. Additionally, because offspring hearts were used for other cardiac studies [27], the Fulton’s Index (RV/LV+Septum) was not performed as a measure of right ventricular hypertrophy associated with persistent PPHN. However, to address this issue, we studied right ventricular sections collected in 4% paraformaldehyde and triple-stained with Alexa Fluor labeled actin-binding phalloidin (green), oligosaccharide binding Wheat Germ Agglutinin (WGA) (red) and 4', 6-diamidino-2-phenylindole (DAPI) counterstain (blue) to delineate cardiomyocyte area. Cross-sections (5 μm) were examined and photographed systematically at 60x using a Nikon P90i microscope (Nikon Instruments Inc., Melville, NY) with a programmable motorized stage and a 100 μm grid centered over the ventricular space. Cardiomyocyte area was measured systematically (3 cells per image) in four regions (anterior, outer and posterior walls and the interventricular septum) using programmable settings for consistency. RV cardiomyocyte area was significantly larger in HF diet-exposed offspring. The effect was significant for diet (p = 0.025) but not diabetes (p = 0.585) or interaction (p = 0.13). Findings correlate with echocardiography and support the fact that a maternal HF diet is associated with PPHN in newborn offspring.

## Conclusions

In conclusion, this study emphasizes the consequences of a maternal HF diet on fetal pulmonary vasculogenesis, demonstrates adverse consequences beyond the perinatal period and directs attention to mechanistic pathways of interest. Knowledge gained provides a foundation for the investigation of preventative and therapeutic strategies aimed at decreasing pulmonary morbidity in at-risk infants. A desperate area in need of ongoing research is to continue to understand how a maternal HF diet could exacerbate the effects of diabetic pregnancy.

## Supporting Information

S1 Table Model characteristics. (DOCX)
