# Supplementary material for: Consequences of a Maternal High-Fat Diet and Late Gestation Diabetes on the Developing Rat Lung
Source: PLoS One. 2016 Aug 12;11(8):e0160818. doi: 10.1371/journal.pone.0160818 (PMC4982689; doi:10.1371/journal.pone.0160818)
Supplement: S1 Table — (DOCX) [file pone.0160818.s001.docx]

**Supplementary Table 1.**  Model characteristics

| Parameter | Controls | Diabetes exposed | Diet exposed | Combination exposed | P value | | | | |
| --- | --- | --- | --- | --- | --- | --- | --- | --- | --- |
|  |  |  |  |  | Diet | Diabetes | | | Interaction |
| **Maternal weight gain (g)** | 28  ±3.64 | 27  ±4.24 | **63**  **±13.89** | **47**  **±8.75** | **≤0.001** | | 0.29 | 0.35 | |
| **Maternal blood glucose (mg/dL)** | 91  ±1.06 | **322**  **±11.1** | 93  ±1.28 | 348  ±12.5 | 0.10 | | **≤0.001** | 0.15 | |
| **Maternal triglyceride (mg/dL)** | 36  ±5.51 | **71**  **±12.50** | **115**  **±20.43** | **555**  **±221** | **0.02** | | **0.04** | 0.08 | |
| **Maternal postpartum leptin (ng/mL)** | 1.40  ±0.21 | 1.06  ±0.17 | **2.90**  **±0.60** | **1.95**  **±0.27** | **≤0.001** | | 0.06 | 0.36 | |
| **Litter size (n)** | 10.67  ±1.12 | 10.85  ±1.16 | 10.73  ±1.23 | 11.09  ±1.60 | 0.91 | | 0.83 | 0.94 | |
| **Offspring insulin (pmol/L)** | 111  ±14 | **468**  **±157** | **387**  **±116** | **1224**  **±367** | **0.01** | | **0.003** | 0.23 | |
| **Offspring triglyceride (mg/dL)** | 93  ±4.29 | 102  ±10.77 | 114  ±15.49 | 91  ±10.53 | 0.60 | | 0.51 | 0.12 | |
| **Offspring IL-6**  **(pg/ml)** ^†^ | 543  ±47 | 481  ±72 | 372  ±29 | ***932**  **±155** |  | |  | **0.006** | |
| **Offspring TNFα (pg/ml)** ^†^ | 28  ±1.96 | 23  ±1.31 | 24  ±1.56 | 32  ±3.85 |  | |  | **0.016** | |

Data presented as means ± SEM is from delivering dams and newborn offspring. Significant differences related to diet or diabetes are by 2-way ANOVA. * Remains significant by one-way ANOVA with Dunnett’s post-test when interaction effect is significant by two-way ANOVA. Significance set at p<0.05. ^†^Data from a subset of newborns pups (n=13-26/group).
